# Immune system dysfunction and inflammation in aging *Shank3b* mutant mice, a model of autism spectrum disorder

**DOI:** 10.3389/fimmu.2024.1447385

**Published:** 2024-09-06

**Authors:** Enrica Cerilli, Ginevra Matilde Dall’O, Gabriele Chelini, Benedetta Catena, Birgit Weinberger, Yuri Bozzi, Luca Pangrazzi

**Affiliations:** ^1^ CIMeC - Center for Mind/Brain Sciences, University of Trento, Rovereto, Trento, Italy; ^2^ Department of Biomedical Sciences, CNR Neuroscience Institute, Pisa, Italy; ^3^ Institute for Biomedical Aging Research, University of Innsbruck, Innsbruck, Austria

**Keywords:** neurodevelopmental disorder, inflammaging, bone marrow, spleen, cerebellum, brain

## Abstract

**Introduction:**

Autism spectrum disorder (ASD) is a heterogeneous group of neurodevelopmental Q8 conditions characterized by deficits in social interaction/communication and restrictive/repetitive behaviors. Recent studies highlight the role of immune system dysfunction and inflammation in ASD pathophysiology. Indeed, elevated levels of pro-inflammatory cytokines were described in the brain and peripheral blood of ASD individuals. Despite this, how this pro-inflammatory profile evolves with aging and whether it may be associated with behavioral deficits is unknown. In this work, we explored the impact of aging on motor behavior and inflammation using Shank3b mutant mice, a model for syndromic ASD.

**Methods:**

Using RT-qPCR and flow cytometry, we examined the expression of key pro-inflammatory molecules in the cerebellum, bone marrow, spleen, and peripheral blood, comparing adult and old *Shank3b* ^+/+^, *Shank3b* ^+/-^, and *Shank3b* ^-/-^ mice.

**Results and discussion:**

Our findings revealed genotype- and age-related differences in inflammation and motor behavior, with *Shank3b^-/-^* mice exhibiting accelerated aging and motor impairments. Correlations between pro-inflammatory molecules and behavioral deficits suggest that a link may be present between systemic inflammation and ASD-related behaviors, underscoring the potential role of age-related inflammation (“inflammaging”) in exacerbating ASD symptoms.

## Introduction

Autism spectrum disorder (ASD) is a heterogenous group of long-term neurodevelopmental disorders characterized by core behavioral symptoms, namely deficits in social interaction and communication as well as repetitive/stereotyped behaviors (1). Alongside with the core symptoms, ASD is associated with multiple neuropsychiatric and neurological conditions with a developmental trajectory including anxiety and depression, epilepsy, language disorders, attention deficit/hyperactivity disorder (ADHD), bipolar disorder, obsessive-compulsive disorder (OCD), and intellectual disability (ID) (2). Besides genetic and environmental factors, immune system dysfunction and neuroinflammation have recently emerged as contributors to ASD pathophysiology. Increased levels of pro-inflammatory cytokines including interleukin (IL)-6, IL-1β, IL-8, tumor necrosis factor (TNF), IL-12p40, and IL-17 were found in the blood and in the brain of ASD children (3–5). A key finding suggesting the presence of impairments in immune system function with ASD was the observation of the enhanced expression of pro-inflammatory markers in post-mortem samples from ASD individuals. These studies described microglia activation and increased pro-inflammatory cytokines and chemokines [such as IL-6, TNF, IL-1β, interferon (IFN)γ, and chemokine C-C motif ligand (CCL)-2] in the brains and cerebrospinal fluid of ASD subjects (6, 7). Immune dysfunction, including the over-activation of immune cells accompanied by increased permeability of the blood–brain barrier, have additionally been reported (8, 9). Mouse models carrying ASD-relevant mutations have been used to assess the contribution of pro-inflammatory processes to ASD (10). In this context, our recent findings reported a wide spectrum of immune system dysfunction and inflammation both in the central nervous system and systemically (11, 12). In particular, pro-inflammatory processes within the cerebellum were associated with ASD-related behaviors in *Cntnap2* mutant mice.

In this study, we took advantage of *Shank3b* mutant mice, an established model of ASD lacking the 3b isoform of the *Shank3* gene (13). SHANK3 codes for the SH3 and multiple ankyrin repeat domain protein 3 (14, 15), which belongs to the family of SHANK proteins and acts as a major scaffolding protein within the postsynaptic density of excitatory neurons (Lim et al., 2013; 16). SHANK3 mutations and deletions are associated with Phelan McDermid Syndrome (PMS), a syndromic form of ASD characterized by intellectual disability, speech and developmental delay, and ASD-related behaviors (17). Importantly, *Shank3b* mutant mice display autistic-like features as social and interaction impairments, decreased locomotor activity as well as sensory processing deficits (13, 18, 19). We recently reported increased levels of molecules related to inflammation and damage in the cerebellum and peripheral blood (PB), as well as a wide immune dysfunction in the bone marrow (BM) and spleen of adult *Shank3b^−/−^* mutant mice (20). These findings strengthen the hypothesis that neuroinflammation and immune system dysfunction may support ASD-like behaviors, thus contributing to the pathogenesis and severity of ASD.

Age-related inflammation (commonly known as “inflammaging”) is a chronic, low-grade inflammation developing during the aging process, which has been associated with the onset of age-related diseases (21). Among all diseases, inflammaging is known to contribute to neurodegenerative disorders including Alzheimer’s and Parkinson’s (22). The over-activation of the innate and adaptive immune responses, as well as cellular senescence and the accumulation of molecules supporting tissue damage, may support inflammaging (21, 23). Despite this, how inflammation and immune dysfunction may progress with aging in the presence of ASD has not been investigated yet.

Here we aimed at gaining insights into the physiopathology of ASD in the context of aging by investigating the interlink between inflammation and ASD-related behaviors in *Shank3b* mice. To this aim, locomotor activity, cerebellar-associated motor coordination as well as engagement in repetitive behaviors were assessed in adult (3-5 months) and old (>18 months) *Shank3b^+/+^*, *Shank3b^+/-^*, and *Shank3b^-/-^* mice. In parallel, immune parameters were assessed in the cerebellum, peripheral blood mononuclear cells (PBMC), bone marrow (BM) and spleen, lymphoid organs responsible for the production and maintenance of immune cells involved in pro-inflammatory processes. A comparison between adult and old mice was made, to shed light on the potential link between pro-inflammatory changes at the systemic level and behavioral phenotypes. Measuring a series of pro-inflammatory and behavioral parameters, we show that *Shank3b^-/-^* mice age more rapidly than their wild-type and heterozygous littermates.

## Results

### Molecules related to inflammation and damage in the cerebellum of *Shank3b* ^+/+^, *Shank3b* ^+/-^, and *Shank3b* ^-/-^ mice

**Figure 1:**
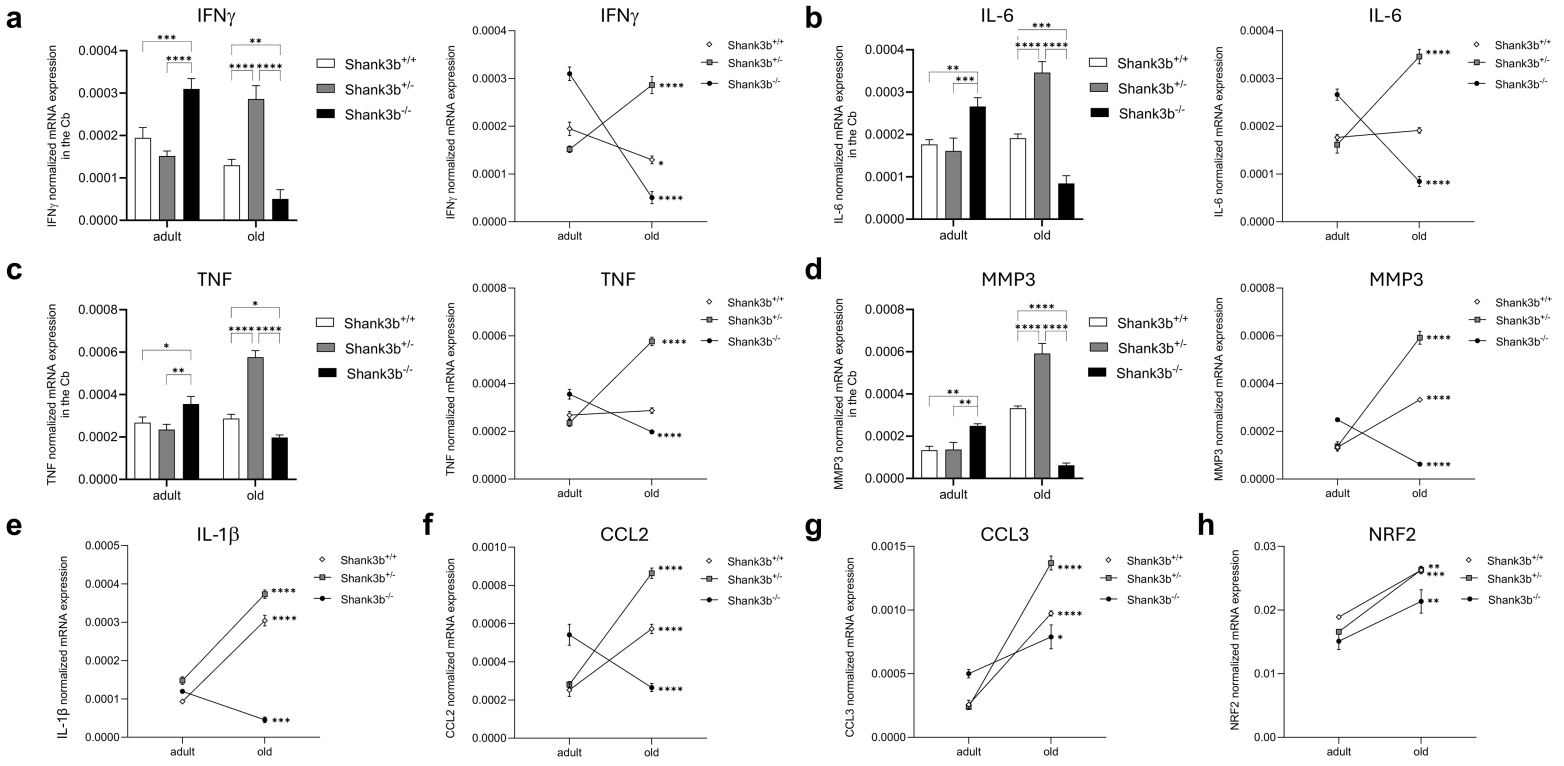
Molecules related to inflammation and damage in the cerebellum of *Shank3b* ^+/+^, *Shank3b* ^+/-^, and *Shank3b* ^-/-^ mice. mRNA expression of **(A)** IFNγ, **(B)** IL-6, **(C)** TNF, **(D)** MMP3, **(E)** IL-1β, **(F)** CCL2, **(G)** CCL3, and **(H)** NRF2 in the cerebellum (Cb) of adult and old *Shank3b^+/+^, Shank3b^+/-^* and *Shank3b^-/-^* mice measured using RT-qPCR. Two-way ANOVA, Tukey *post-hoc* test. n = 8 per group. *p<0.05; **p<0.01; ***p<0.001; ****p<0.0001.

Our recent study documented pro-inflammatory changes in the cerebellum of adult *Shank3b* ^-/-^ mice (20). Thus, we assessed whether the mRNA expression of pro-inflammatory molecules may change during aging in the cerebellum of *Shank3b* ^+/+^, *Shank3b* ^+/-^, and *Shank3b* ^-/-^ mice (**Figure 1**). In adult mice, the levels of IFNγ, IL-6, TNF, and MMP3 were similar between *Shank3b* ^+/+^ and *Shank3b* ^+/-^ mice while they increased in *Shank3b* ^-/-^ animals (**Figures 1A-D**). Intriguingly, in the old group, the expression of all four molecules was the highest in *Shank3b* ^+/-^, intermediate in *Shank3b* ^+/+^ and the lowest in *Shank3b* ^-/-^ mice. Overall, the production of molecules related to inflammation and damage was stable during aging in *Shank3b* ^+/+^ mice (with the exception of IFNγ, which decreased), while it was reduced in the *Shank3b* ^-/-^ group. Intriguingly, IFNγ, IL-6, TNF, and MMP3 were strongly upregulated during aging in the cerebellum of *Shank3b* ^+/-^ animals. Similarly, IL-1β and CCL2 expression increased with age in *Shank3b* ^+/+^ and *Shank3b* ^+/-^ mice, but it was reduced in *Shank3b* ^-/-^ animals (**Figures 1E, F**). In parallel, the levels of CCL3 and NRF2 increased during aging in all groups (**Figures 1G, H**). In particular, NRF2 has been shown to be induced by oxidative stress (24). Taken together, the expression of molecules related to inflammation and damage differentially change during aging in *Shank3b* ^+/+^, *Shank3b* ^+/-^, and *Shank3b* ^-/-^ mice.

### Molecules related to inflammation and damage in the bone marrow and spleen of *Shank3b* ^+/+^, *Shank3b* ^+/-^, and *Shank3b* ^-/-^ mice

**Figure 2:**
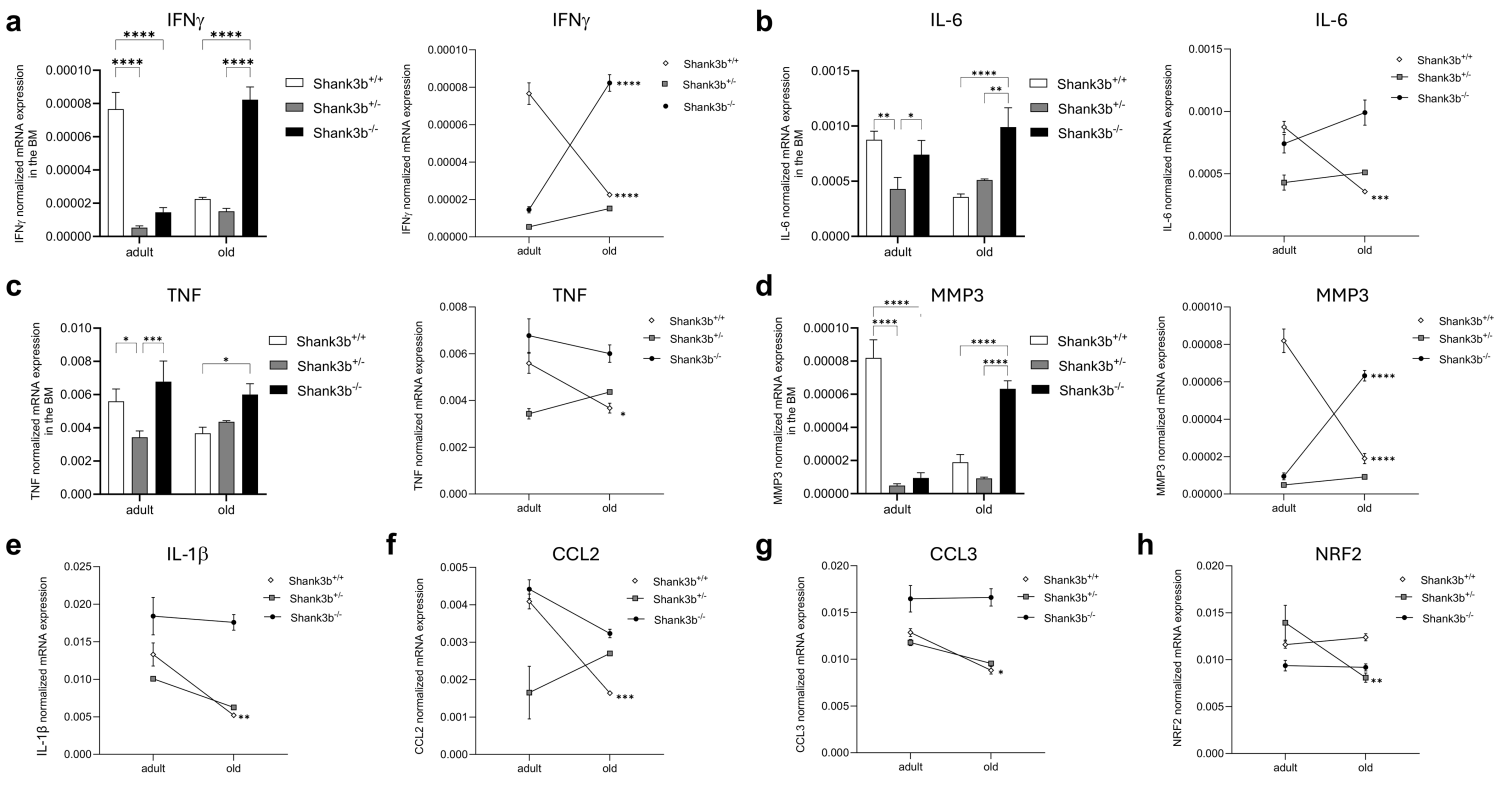
Molecules related to inflammation and damage in the bone marrow of *Shank3b* ^+/+^, *Shank3b* ^+/-^, and *Shank3b* ^-/-^ mice. mRNA expression of **(A)** IFNγ, **(B)** IL-6, **(C)** TNF, **(D)** MMP3, **(E)** IL-1β, **(F)** CCL2, **(G)** CCL3, and **(H)** NRF2 in the bone marrow (BM) of adult and old *Shank3b^+/+^, Shank3b^+/-^* and *Shank3b^-/-^* mice measured using RT-qPCR. Two-way ANOVA, Tukey *post-hoc* test. n = 8 per group. *p<0.05; **p<0.01; ***p<0.001; ****p<0.0001.

To assess whether the situation in the cerebellum may be paralleled by changes in the bone marrow (BM), the mRNA expression of pro-inflammatory cytokines and molecules related to damage and oxidative stress was measured in BM cells from *Shank3b* ^+/+^, *Shank3b* ^+/-^, and *Shank3b* ^-/-^ mice (**Figure 2**). Differently from the cerebellum, IFNγ levels in adult mice were higher in *Shank3b* ^+/+^ mice when compared to the other two groups (**Figure 2A**). Despite this, the expression of this molecule strongly increased with aging in *Shank3b* ^-/-^ mice, while in the *Shank3b* ^+/+^ group it was decreased and comparable with their *Shank3b* ^+/-^ counterpart. In parallel, IL-6 and TNF were expressed at a similar level in adult *Shank3b* ^+/+^ and *Shank3b* ^-/-^ mice, while they were both reduced in the adult *Shank3b* ^+/-^ group. Again, the levels of these cytokines were reduced with age in *Shank3b* ^+/+^ and increased in *Shank3b* ^-/-^ mice, but the expression was stable in *Shank3b* ^+/-^ animals (**Figures 2B, C**). Similarly, MMP3 expression was reduced with aging in *Shank3b* ^+/+^ mice, increased in *Shank3b* ^-/-^ and did not change in the *Shank3b* ^+/-^ group (**Figure 2D**). Comparable results were obtained when the expression of IL-1β, CCL2 and CCL3 was assessed, although in this case an age-related decrease was described in *Shank3b* ^+/+^ mice but no significant differences were documented for both *Shank3b* ^+/-^ and *Shank3b* ^-/-^ mice (**Figures 2E–G**). Finally, NRF2 was reduced in old when compared to adult *Shank3b* ^-/-^ animals but it was not affected by aging in the other groups (**Figure 2H**).

**Figure 3:**
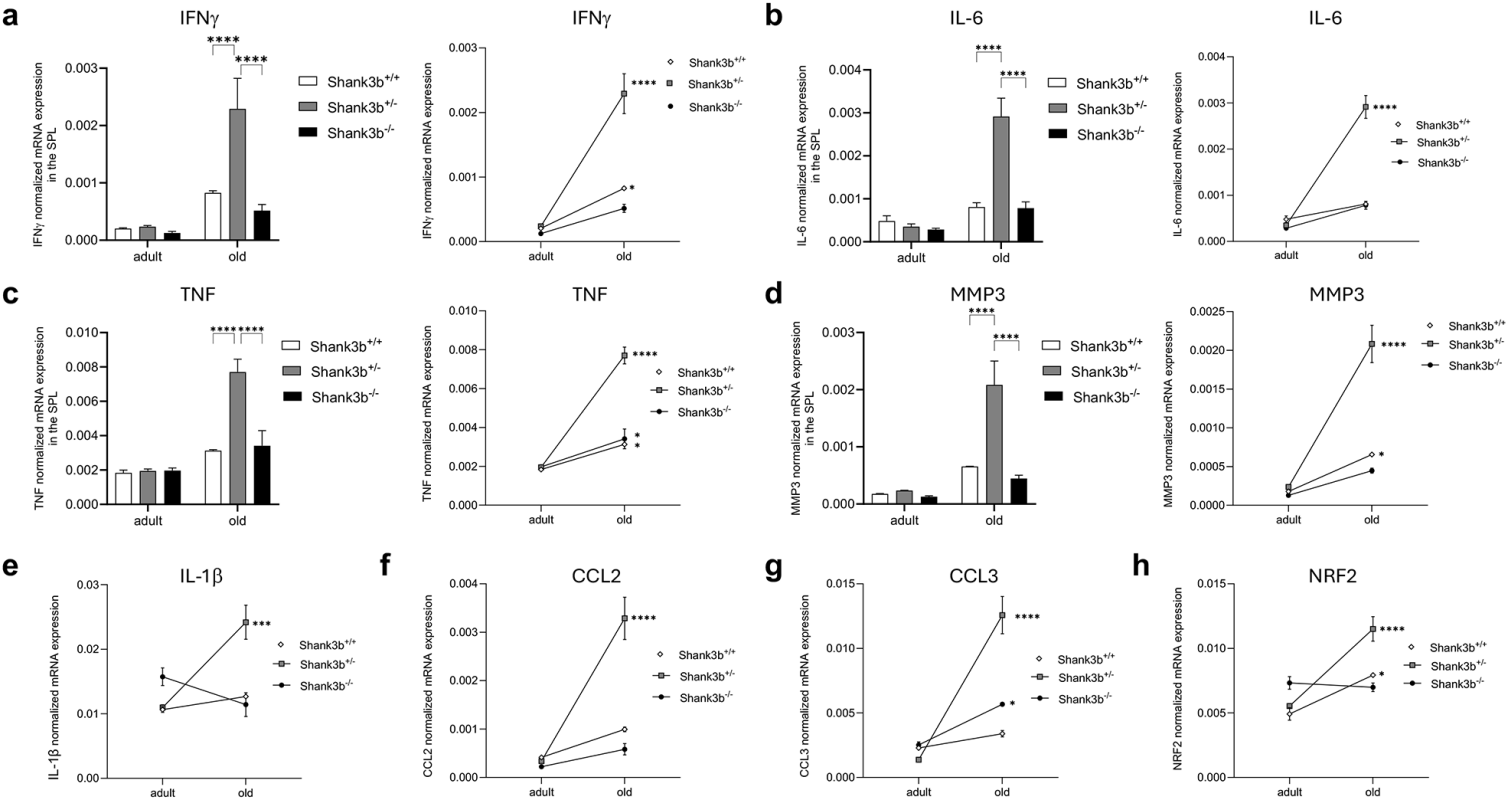
Molecules related to inflammation and damage in the spleen of *Shank3b* ^+/+^, *Shank3b* ^+/-^, and *Shank3b* ^-/-^ mice. mRNA expression of **(A)** IFNγ, **(B)** IL-6, **(C)** TNF, **(D)** MMP3, **(E)** IL-1β, **(F)** CCL2, **(G)** CCL3, and **(H)** NRF2 in the spleen of adult and old *Shank3b^+/+^, Shank3b^+/-^* and *Shank3b^-/-^* mice measured using RT-qPCR. Two-way ANOVA, Tukey *post-hoc* test. n = 8 per group. *p<0.05; *** p<0.001; ****p<0.0001.

Next, the mRNA expression of the same molecules measured in the cerebellum and BM was assessed in the spleen (**Figure 3**). Overall, no significant differences between *Shank3b* ^+/+^, *Shank3b* ^+/-^, and *Shank3b* ^-/-^ mice were found when adult mice were compared (**Figures 3A–D**). In old age, the expression of IFNγ, IL-6, TNF, and MMP3 was higher in the *Shank3b* ^+/-^ group in relationship with *Shank3b* ^+/+^ and *Shank3b* ^-/-^ animals, although no changes between these two groups were observed. Furthermore, the levels of all molecules increased with age in the spleen of *Shank3b* ^+/-^ mice, although some were additionally overexpressed in old *Shank3b* ^+/+^ and *Shank3b* ^-/-^ in comparison with their adult counterpart. Similarly, a strong increase in the expression of IL-1β, CCL2, CCL3 and NRF2 was described in the spleen of old *Shank3b* ^+/-^ mice (**Figures 3E–H**). In summary, genotype- and age-related changes in the expression of pro-inflammatory molecules are present in the BM and in the spleen of *Shank3b* mice.

### Pro-inflammatory phenotype changes drastically with aging in a tissue-specific and genotype-dependent fashion

**Figure 4:**
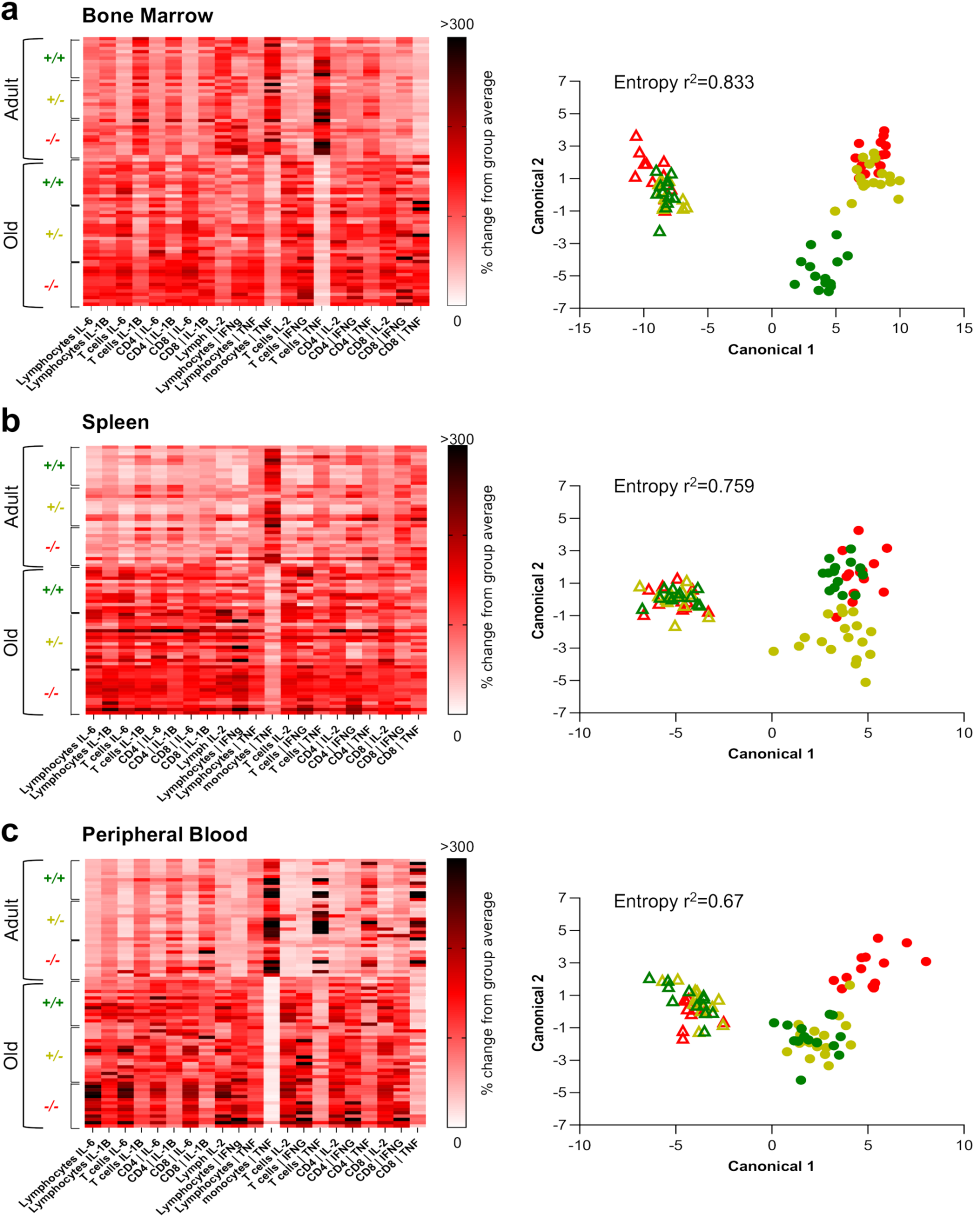
The expression pattern of pro-inflammatory cytokines varies with age, according to a genotype- and tissue- dependent logic. a-c, left panels) Heatmaps showing the mean fluorescence cytokine intensity within lymphocytes, CD14^+^ cells, T cells, CD8^+^ and CD4^+^ T cells (defined as shown in **Supplementary Figure S1**) of adult and old *Shank3b^+/+^, Shank3b^+/-^* and *Shank3b^-/-^* mice in the bone marrow, spleen and peripheral blood. **(A-C)**, right panels) Scatter plot showing the clustering of the six experimental groups obtained with the discriminant analysis. Two-way ANOVA, Tukey *post-hoc* test. Adult mice (shown with triangles): n=12 (*Shank3b^+/+^*), n=12 (*Shank3b^+/^*)*^-^*, n=12 (*Shank3b^-/-^*). Old mice (shown with dots): n=14 (*Shank3b^+/+^*), n=18 (*Shank3b^+/^*)*^-^*, n=14 (*Shank3b^-/-^*). Comparisons with p<0.05 are reported in green in the right panels.

To recapitulate our findings and achieve a qualitative understanding of the pro-inflammatory state of peripheral tissues across experimental conditions, we used mathematical modelling to cluster the pro-inflammatory profile of the six groups (namely, adult and old *Shank3b* ^+/+^, *Shank3b* ^+/-^, and *Shank3b* ^-/-^ mice) based on their cytokine expression assessed using flow cytometry (**Figure 4**). The gating strategy used to define the populations of interest is shown in **Supplementary Figure S1**. First, we observed a clear separation between adult and aged mice in the expression pattern of pro-inflammatory cytokines in all tissue analyzed (**Figures 4A–C**). Key differences were statistically confirmed using a two-way ANOVA on the mahalanobis distances of each subject as a measure of proximity to the center of mass of each cluster (**Supplementary Figure S2**). Conversely, genotype-driven differences were presented with a tissue-dependent logic. In adult mice, statistically significant differences were only found between *Shank3b^+/+^* and *Shank3b^-/-^* animals in the BM, whereas other tissues showed similar inflammatory phenotype. In the BM of old mice, both partial and full deletion of *Shank3b* resulted in a significant and similar change compared to *Shank3b^+/+^* animals (**Figure 4A**; **Supplementary Figure S2A**). Similarly, both the old *Shank3b* ^+/-^ and *Shank3b^-/-^* groups were significantly distanced with respect to *Shank3b^+/+^* animals in the spleen, but not vice-versa (**Figure 4B**; **Supplementary Figure S2B**). Finally, in the PB, full deletion of *Shank3b* determined a unique pro-inflammatory phenotype in aged mice, while no differences were observed in old mice with heterozygous mutation when compared with their *Shank3b^+/+^* counterpart (**Figure 4C**; **Supplementary Figure S2C**). Taken together, distinct pro-inflammatory profiles may be present in adult and old *Shank3b^+/+^*, *Shank3b* ^+/-^, and *Shank3b^-/-^* mice, with prominent age-related differences.

### Motor behavior in *Shank3b* ^+/+^, *Shank3b* ^+/-^, and *Shank3b* ^-/-^ mice

**Figure 5:**
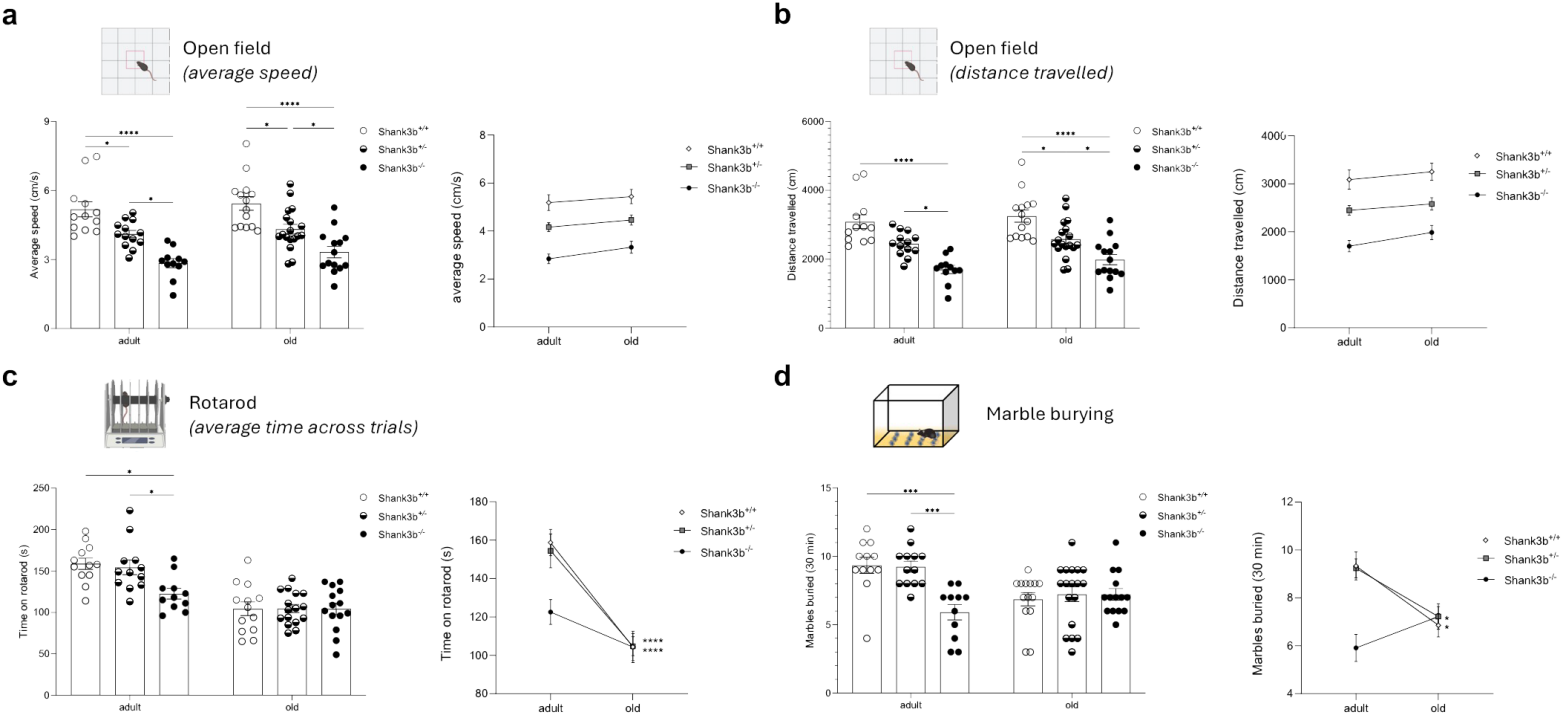
Motor behavior in *Shank3b* ^+/+^, *Shank3b* ^+/-^, and *Shank3b* ^-/-^ mice. Average speed (cm/s) **(A)** and distance travelled **(B)** in the open field test in adult and old *Shank3b^+/+^*, *Shank3b^+/-^* and *Shank3b^-/-^* mice. **(B)** Average time on rotarod (latency to fall, s) in the rotarod test. **(C)** Number of marbles buried in the marble burying test. Two-way ANOVA, Tukey *post-hoc* test. Adult mice: n=12 (*Shank3b^+/+^*), n=12 (*Shank3b^+/^*)*^-^*, n=12 (*Shank3b^-/-^*). Old mice: n=14 (*Shank3b^+/+^*), n=18 (*Shank3b^+/^*)*^-^*, n=14 (*Shank3b^-/-^*). *p<0.05; *** p<0.001; ****p<0.0001.

Alongside with the investigations at the molecular level, we assessed motor behaviors in adult and old *Shank3b* ^+/+^, *Shank3b* ^+/-^, and *Shank3b* ^-/-^ mice (**Figure 5**). At both ages, average speed and distance travelled in the open field test were the highest in *Shank3b* ^+/+^, intermediate in *Shank3b* ^+/-^ and the lowest in *Shank3b* ^-/-^ animals (**Figures 5A, B**). Despite this, no age-related differences could be described. The rotarod test is commonly used to assess motor function and coordination of rodents (25). In the adult group, the time spent on the rotarod was decreased in *Shank3b* ^-/-^ in comparison with *Shank3b* ^+/+^ and *Shank3b* ^+/-^ mice (**Figure 5C**). No differences could be observed in old animals, although the time on the rotarod was reduced during aging in both *Shank3b* ^+/+^ and *Shank3b* ^+/-^ mice. Furthermore, mice were subjected to marble burying test, which evaluates repetitive behaviors but also object-oriented perseveration (26, 27). Reduced numbers of marbles buried were observed in adult *Shank3b* ^-/-^ mice in comparison to the other two age-matched groups, while no differences were present in old age. Similarly to the rotarod test, decreased performances were described during aging in *Shank3b* ^+/+^ and *Shank3b* ^+/-^ mice. Taken together, motor impairments are present in *Shank3b* ^-/-^ mice already from adulthood, and they additionally develop in *Shank3b* ^+/+^ and *Shank3b* ^+/-^ animals in old age.

### Relationship between pro-inflammatory molecules in the BM, spleen, PB and cerebellum and motor behavior

**Figure 6:**
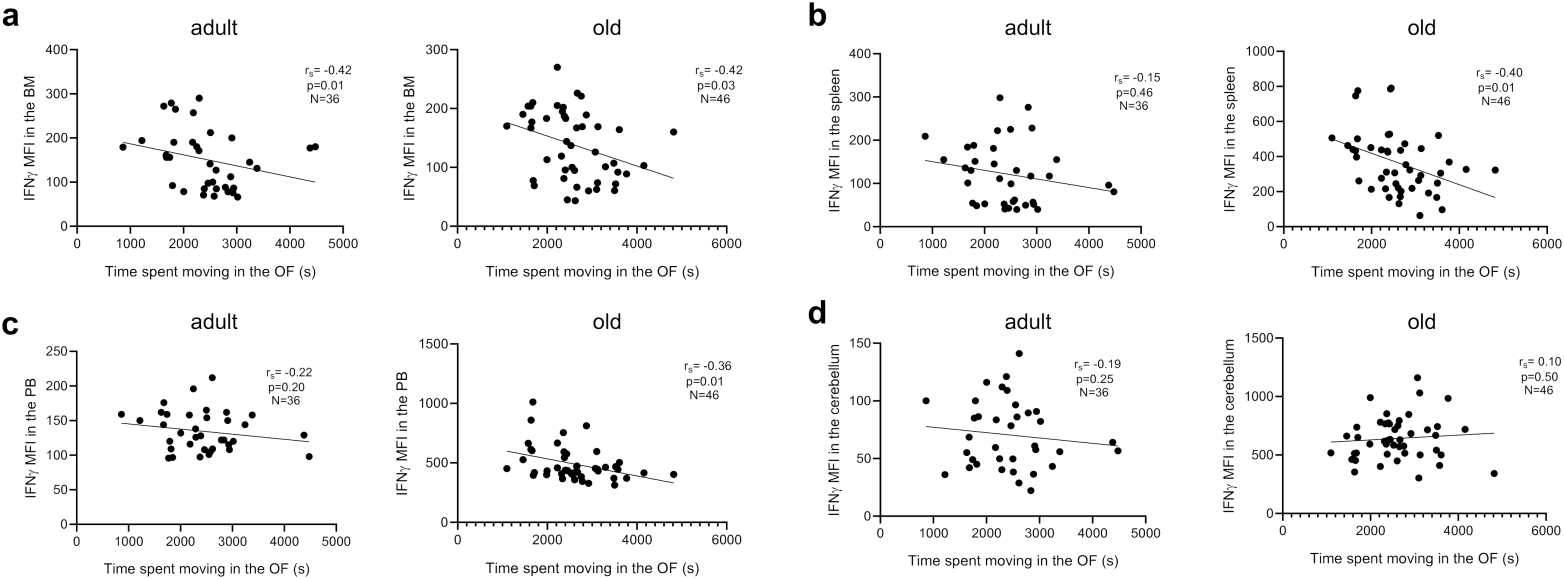
Relationship between pro-inflammatory molecules in the BM, spleen, PB and cerebellum and motor behavior. Correlation between IFNγ mean fluorescence intensity (MFI) and time spent moving in the open field (OF) test in adult and old mice in **(A)** BM, **(B)** spleen, **(C)** PB, and **(D)** cerebellum. Spearman correlation, adult mice n=36, old mice n= 46. Spearman correlation coefficient (r_S_) and p values are shown in the graphs.

We next assessed whether motor behaviors in *Shank3b* ^+/+^, *Shank3b* ^+/-^, and *Shank3b* ^-/-^ mice may be related with the expression of pro-inflammatory molecules (**Figure 6** and **Tables 1**–**4**). In adult mice, the expression of pro-inflammatory molecules in the BM negatively correlated with the distance moved in the open field test (**Figure 6A**) and the average time spent on rotarod (**Table 1**), independently of genotype. Furthermore, the number of marbles buried was negatively correlated with the expression of IFNγ, IL-6, and IL-1β in adult mice (**Table 1**). Similar results were found for the old group, although in this case the negative correlations with the distance moved were stronger but no connection could be described between BM cytokines and average time on rotarod (**Figure 6A**; **Table 1**). In addition, in old age, the expression of IFNγ and IL-1β positively correlated with the numbers of buried marbles (**Table 1**).

**Table 1:** Correlation between behavior and inflammation in the BM of adult/old *Shank3b* mice.

OF distance moved vs	adult		old	
	r_s_	p value	r_s_	p value
IFNγ all cells	**-0.42**	**0.011**	**-0.42**	**0.003**
IFNγ T cells	**-0.40**	**0.032**	-0.40	0.005
TNF all cells	-0.09	0.59	**-0.62**	**<0.0001**
TNF T cells	-0.27	0.22	-0.14	0.37
TNF CD14^+^ cells	0.11	0.52	**-0.47**	**<0.0001**
IL-6 all cells	-0.26	0.13	-0.18	0.23
IL-6 T cells	**-0.34**	**0.04**	-0.19	0.18
IL-6 CD14+ cells	-0.20	0.28	**-0.38**	**0.001**
IL-1β all cells	-0.30	0.07	-0.24	0.12
IL-1β T cells	-0.24	0.15	**-0.32**	**0.03**
IL-1β CD14^+^ cells	-0.20	0.25	**-0.37**	**0.01**
Rotarod average time vs	adult		old	
	r_s_	p value	r_s_	p value
IFNγ all cells	**-0.54**	**0.0002**	0.17	0.24
IFNγ T cells	**-0.36**	**0.03**	0.03	0.63
TNF all cells	0.07	0.67	-0.002	0.99
TNF T cells	-0.10	0.54	0.21	0.15
TNF CD14^+^ cells	-0.17	0.64	0.03	0.46
IL-6 all cells	-0.05	0.78	0.09	0.72
IL-6 T cells	-0.04	0.80	0.14	0.36
IL-6 CD14^+^ cells	-0.01	0.95	0.02	0.82
IL-1β all cells	**-0.34**	**0.04**	0.11	0.65
IL-1β T cells	**-0.39**	**0.02**	0.10	0.49
IL-1β CD14^+^ cells	-0.19	0.06	0.12	0.43
Buried marbles vs	adult		old	
	r_s_	p value	r_s_	p value
IFNγ all cells	**-0.37**	**0.03**	0.22	0.13
IFNγ T cells	-0.21	0.20	0.06	0.67
TNF all cells	-0.27	0.09	-0.05	0.69
TNF T cells	0.007	0.96	**-0.30**	**0.04**
TNF CD14^+^ cells	0.024	0.92	0.14	0.46
IL-6 all cells	**-0.54**	**0.0006**	**0.36**	**0.02**
IL-6 T cells	**-0.35**	**0.03**	**0.29**	**0.05**
IL-6 CD14^+^ cells	-0.28	0.09	0.09	0.86
IL-1β all cells	-0.28	0.10	**0.30**	**0.04**
IL-1β T cells	**-0.38**	**0.03**	-0.04	0.79
IL-1β CD14^+^ cells	-0.13	0.46	0.12	0.81

Statistically significant values are displayed in bold and underlined.

**Table 2:** Correlation between behavior and inflammation in the spleen of adult/old *Shank3b* mice.

OF distance moved vs	adult		old	
	r_s_	p value	r_s_	p value
IFNγ all cells	-0.15	0.46	**-0.40**	**0.01**
IFNγ T cells	-0.12	0.49	**-0.35**	**0.03**
TNF all cells	-0.04	0.80	**-0.38**	**0.006**
TNF T cells	-0.06	0.53	**-0.37**	**0.01**
TNF CD14^+^ cells	-0.09	0.61	**-0.49**	**0.0006**
IL-6 all cells	**-0.38**	**0.02**	-0.16	0.29
IL-6 T cells	**-0.33**	**0.05**	-0.007	0.96
IL-6 CD14^+^ cells	**-0.28**	**0.09**	-0.20	0.25
IL-1β all cells	**-0.34**	**0.04**	**-0.34**	**0.02**
IL-1β T cells	-0.27	0.10	**-0.39**	**0.007**
IL-1β CD14^+^ cells	-0.28	0.10	**-0.33**	**0.03**
Rotarod average time vs	adult		old	
	r_s_	p value	r_s_	p value
IFNγ all cells	**-0.32**	**0.05**	-0.10	0.47
IFNγ T cells	**-0.36**	**0.03**	0.02	0.86
TNF all cells	0.21	0.21	**-0.32**	**0.03**
TNF T cells	0.05	0.74	**-0.32**	**0.03**
TNF CD14^+^ cells	0.16	0.34	-0.16	0.28
IL-6 all cells	-0.24	0.15	-0.18	0.21
IL-6 T cells	-0.20	0.35	-0.06	0.65
IL-6 CD14^+^ cells	-0.25	0.12	-0.038	0.87
IL-1β all cells	-0.25	0.13	0.02	0.99
IL-1β T cells	**-0.38**	**0.02**	-0.17	0.29
IL-1β CD14^+^ cells	**-0.34**	**0.04**	0.13	0.40
Buried marbles vs	adult		old	
	rs	p value	rs	p value
IFNγ all cells	**-0.38**	**0.02**	0.08	0.59
IFNγ T cells	**-0.37**	**0.03**	0.05	0.73
TNF all cells	0.04	0.82	0.09	0.57
TNF T cells	0.05	0.74	0.09	0.54
TNF CD14^+^ cells	-0.02	0.73	0.17	0.26
IL-6 all cells	-0.24	0.16	0.28	0.05
IL-6 T cells	-0.22	0.16	0.27	0.07
IL-6 CD14^+^ cells	-0.28	0.09	0.22	0.15
IL-1β all cells	-0.26	0.12	0.22	0.15
IL-1β T cells	**-0.34**	**0.04**	0.24	0.10
IL-1β CD14^+^ cells	**-0.32**	**0.04**	0.06	0.70

Statistically significant values are displayed in bold and underlined.

**Table 3:** Correlation between behavior and inflammation in the blood of adult/old *Shank3b* mice.

OF distance moved vs	adult		old	
	r_s_	p value	r_s_	p value
IFNγ all cells	-0.22	0.20	**-0.36**	**0.01**
IFNγ T cells	0.09	0.60	**-0.47**	**0.001**
TNF all cells	-0.04	0.82	**-0.54**	**<0.0001**
TNF T cells	-0.007	0.97	**-0.31**	**0.04**
TNF CD14^+^ cells	-0.002	0.99	-0.003	0.95
IL-6 all cells	-0.30	0.07	-0.17	0.24
IL-6 T cells	-0.14	0.41	**-0.46**	**0.0012**
IL-6 CD14^+^ cells	-0.06	0.74	-0.0013	0.87
IL-1β all cells	-0.20	0.23	-0.04	0.81
IL-1β T cells	-0.19	0.27	**0.36**	**0.01**
IL-1β CD14^+^ cells	**-0.34**	**0.004**	0.19	0.25
Rotarod average time vs	adult		old	
	r_s_	p value	r_s_	p value
IFNγ all cells	-0.23	0.17	0.20	0.17
IFNγ T cells	-0.03	0.86	0.14	0.37
TNF all cells	0.28	0.10	-0.08	0.58
TNF T cells	0.27	0.10	-0.13	0.38
TNF CD14^+^ cells	0.21	0.21	-0.12	0.42
IL-6 all cells	0.01	0.94	0.03	0.83
IL-6 T cells	0.05	0.75	0.03	0.84
IL-6 CD14^+^ cells	0.03	0.84	0.08	0.74
IL-1β all cells	0.02	0.87	0.17	0.24
IL-1β T cells	0.16	0.34	**0.29**	**0.05**
IL-1β CD14^+^ cells	-0.30	0.07	0.07	0.66
Buried marbles vs	adult		old	
	rs	p value	rs	p value
IFNγ all cells	-0.30	0.07	0.04	0.79
IFNγ T cells	-0.23	0.13	0.01	0.92
TNF all cells	0.15	0.38	0.10	0.46
TNF T cells	0.24	0.20	0.13	0.39
TNF CD14^+^ cells	0.21	0.25	-0.05	0.76
IL-6 all cells	-0.28	0.09	**0.32**	**0.03**
IL-6 T cells	**-0.38**	**0.02**	0.24	0.10
IL-6 CD14^+^ cells	**-0.34**	**0.04**	0.26	0.08
IL-1β all cells	-0.29	0.08	0.18	0.23
IL-1β T cells	-0.14	0.42	0.10	0.49
IL-1β CD14^+^ cells	**-0.38**	**0.02**	0.25	0.09

Statistically significant values are displayed in bold and underlined.

**Table 4:** Correlation between behavior and inflammation in the cerebellum of adult/old *Shank3b* mice.

OF distance moved vs	adult		old	
	r_s_	p value	r_s_	p value
IFNγ all cells	-0.19	0.25	0.10	0.50
IFNγ T cells	-0.11	0.50	0.27	0.07
TNF all cells	0.089	0.60	0.21	0.17
TNF T cells	0.17	0.31	0.08	0.57
TNF CD14^+^ cells	0.14	0.42	0.01	0.91
IL-6 all cells	-0.15	0.38	0.19	0.20
IL-6 T cells	-0.26	0.13	-0.10	0.59
IL-6 CD14^+^ cells	-0.21	0.22	0.10	0.45
IL-1β all cells	0.05	0.75	**0.35**	**0.02**
IL-1β T cells	-0.05	0.76	0.12	0.52
IL-1β CD14^+^ cells	-0.02	0.93	0.28	0.12
Rotarod average time vs	adult		old	
	r_s_	p value	r_s_	p value
IFNγ all cells	0.16	0.35	0.05	0.73
IFNγ T cells	0.12	0.48	0.12	0.44
TNF all cells	0.20	0.23	-0.01	0.87
TNF T cells	0.25	0.14	0.27	0.07
TNF CD14^+^ cells	-0.24	0.16	0.24	0.12
IL-6 all cells	0.18	0.28	0.05	0.74
IL-6 T cells	0.09	0.57	-0.14	0.38
IL-6 CD14^+^ cells	-0.21	0.22	-0.02	0.85
IL-1β all cells	0.05	0.73	0.17	0.2
IL-1β T cells	0.03	0.84	0.08	0.68
IL-1β CD14^+^ cells	-0.14	0.41	0.12	0.44
Buried marbles vs	adult		old	
	r_s_	p value	r_s_	p value
IFNγ all cells	0.006	0.97	0.22	0.13
IFNγ T cells	0.12	0.53	0.044	0.76
TNF all cells	0.11	0.50	-0.05	0.73
TNF T cells	0.10	0.57	0.05	0.74
TNF CD14^+^ cells	-0.24	0.16	0.001	0.99
IL-6 all cells	0.09	0.59	**0.29**	**0.05**
IL-6 T cells	-0.02	0.46	0.09	0.53
IL-6 CD14^+^ cells	-0.29	0.08	0.18	0.17
IL-1β all cells	0.10	0.55	**0.35**	**0.02**
IL-1β T cells	-0.18	0.3	0.15	0.34
IL-1b CD14^+^ cells	-0.18	0.22	**0.29**	**0.04**

Statistically significant values are displayed in bold and underlined.

In the spleen, pro-inflammatory cytokines were weakly related to parameters of motor behavior in adult mice, although IL-6 and IL-1β showed significant negative correlations with buried marbles (**Figure 6B**; **Table 2**), again independently of genotype. In old mice, a negative relationship was observed between IFNγ, TNF, IL-1β, and distance moved in the open field (**Figure 6B**), and TNF expression with average time on rotarod (**Table 2**). Similar results were observed in the PB for adult mice (**Figure 6C**; **Table 3**). In the old group, while the expression of IFNγ, TNF, and IL-6 negatively correlated with the distance moved in the open field (**Figure 6C**; **Table 3**), positive correlations were observed between IL-1β levels and distance moved in the open field or average time on the rotarod (**Table 3**). Furthermore, IL-6 levels positively correlated with the number of marbles buried (**Table 3**).

Finally, the levels of pro-inflammatory molecules in the cerebellum did not correlate with any parameters of the behavioral test in the adult group (**Figure 6D**; **Table 4**). Despite this, a positive correlation between IL-1β expression and distance moved in the open field, as well as IL-1β and IL-6 levels and marble buried, could be observed in old animals (**Table 4**), independently of genotype.

In summary, our results indicate that pro-inflammatory molecules in the BM, spleen, PB and cerebellum correlated with parameters of behavioral tests in both adult and old mice.

## Discussion

ASD is a complex neurodevelopmental disorder with significant clinical and etiological heterogeneity. Previous studies have found increased levels of pro-inflammatory molecules and immune system dysfunction in individuals with ASD as well as in mouse models (11, 28). In particular, our recent work performed using adult animals described that molecules related to inflammation and damage were increased in the cerebellum of two mouse models of ASD (12, 20). Despite this, no studies have investigated how ASD progresses with aging at both molecular and behavioral levels, neither in humans nor in mouse models. A key unexplored question is whether the aging process in the presence of ASD exhibits similarities or differences compared to typical “healthy” aging. Inflammaging is known to play a determinant role in the pathogenesis of age-related diseases and in the aging process itself (21). In particular, chronic diseases can be considered a manifestation of accelerated aging. Inflammaging represents a highly significant risk factor for the development of neurodegenerative diseases including Alzheimer’s and Parkinson’s, which are characterized by progressive degeneration of neurons in the brain and peripheral nervous system, and they are additionally accompanied by immune system dysfunction (22, 29). Considering the presence of pro-inflammatory impairments with ASD, it is intriguing to unravel how inflammaging takes place in individuals suffering from this neurodevelopmental disorder and whether this condition may further exacerbate behavioral impairments Furthermore, it is fundamental to understand whether a connection may exist between expression of pro-inflammatory molecules at the systemic level and behavioral deficits.

In this study, we assessed how pro-inflammatory molecules change during aging in the cerebellum, BM, spleen and PB of *Shank3b* ^+/+^, *Shank3b* ^+/-^, and *Shank3b* ^-/-^ mice. *Shank3b* ^-/-^ mice represent an established mouse model of syndromic ASD (13). Among the syndromic forms of autism, Phelan-McDermid syndrome (PMS) is a rare neurodevelopmental disorder characterized by autistic-like behaviors, developmental delay, intellectual disability, sensory processing dysfunction and poor motor function (17). Deletion or mutations of the SHANK3 gene, coding for SH3 and multiple ankyrin repeat domains protein 3, cause PMS (15). Accordingly, mice lacking *Shank3b* recapitulate typical autism-like symptoms, including social interaction and communication deficits (13), aberrant sensory processing (18, 30), and prominent motor dysfunction (31). As SHANK3 mutations in humans are often present in heterozygosis, a group of *Shank3^+/-^* mice was additionally considered for this work.

In accordance with our previous study (20), the levels of pro-inflammatory molecules were increased in the cerebellum of adult *Shank3b* ^-/-^ mice, while no differences were observed between the *Shank3b* ^+/+^ and *Shank3b* ^+/-^ animals (**Figure 1**). Intriguingly, the effects of aging on cerebellar inflammation were different in the three groups. While typical signs of inflammaging could be observed in *Shank3b* ^+/+^ mice and they were even more pronounced in *Shank3b* ^+/-^ animals, the expression of pro-inflammatory molecules decreased with age in the *Shank3b* ^-/-^ group. Although this last result may be counterintuitive, we hypothesize that in *Shank3b* ^-/-^ mice the functionality of cerebellum may be so compromised that the production of pro-inflammatory molecules is almost completely blocked. We recently described a similar situation for the cerebellum of *Engrailed 2* (*En2^-/-^*) mice, another mouse model of ASD displaying severe neuroanatomical impairments paralleled by decreased expression of pro-inflammatory molecules (11). This situation seems to be different for *Shank3b* ^+/-^ animals, in which mild deficits may lead to increased age-related inflammation in the cerebellum. To confirm this hypothesis, future studies must characterize whether neuroanatomical impairments may additionally be present in old *Shank3b* ^-/-^ animals. Importantly, we speculate that microglia may regulate cerebellar pro-inflammatory processes, and thus this cell type may be impaired in old *Shank3b^-/-^* mice. In our work, the expression of pro-inflammatory markers was not measured directly within microglia cells but in CD14^+^ cells, innate immune cell type including also microglia cells (32). Indeed, expression of microglia marker Tmem119 is known to be downregulated with stimulation (33), and thus it was not possible to define directly this cell type in our assay. Despite this, evidence collected in our recent study performed in another mouse model of ASD (namely *Cntnap2^-/-^* mice) suggests that microglia may play a key role in pro-inflammatory processes in the cerebellum and in the onset of ASD-related behaviors.

A partially different situation was observed for the BM, in which the mRNA expression of pro-inflammatory molecules in adult mice was the highest in *Shank3b* ^+/+^ mice (**Figure 2**), although less differences were observed when cytokines were assessed at the protein level after stimulation (**Figure 4A**). Furthermore, molecules related to inflammation decreased with aging in the *Shank3b* ^+/+^ group, were stable in *Shank3b* ^+/-^ and increased in *Shank3b* ^-/-^ mice. This may be caused by impaired activation capacity and functionality of immune cells with aging in *Shank3b* ^+/+^ mice which may keep inflammaging under control. Despite this, age-related inflammation could be observed in the BM of *Shank3b* ^-/-^ animals. In the spleen, pro-inflammatory molecules were highly increased in *Shank3b* ^+/-^ mice, although signs of inflammaging were present also in the other two groups. Discriminant analysis performed on flow cytometry data showed that the BM may be particularly sensitive to *Shank3b* deletion (**Figure 4**), as changes were reported in adult mice with full deletion (*Shank3b* ^-/-^), and in both *Shank3b* ^+/-^ and *Shank3b* ^-/-^ in old age. Conversely, *Shank3b* mutation showed limited impact on pro-inflammatory phenotype in the spleen. Finally, in the PB, a specific effect of Shank3b deletion emerged exclusively in old age.

To investigate motor behavior and coordination, open field and rotarod tests were performed on all groups (**Figure 5**). At both ages, mild and severe motor impairments (assessed using the open field test) could be described respectively in *Shank3b^+/-^* and *Shank3b^-/-^* mice, although no age-related changes were found. Conversely, motor coordination in *Shank3b* ^+/+^ and *Shank3b^+/-^* mice was reduced with aging at the level of *Shank3b^-/-^* animals. The Marble burying test is usually performed to investigate the presence of repetitive behaviors in mice (26). Nonetheless, adult *Shank3b^-/-^* mice buried less marbles in comparison with the other groups. Although counterintuitive, this data is perfectly in line with previous studies performed in other Shank3 mutant models (19, 34, 35). As proposed by Kouser and colleagues, the fact that *Shank3b^-/-^* mice did not show interest in burying marbles can be due to an exhibition of an avoidance phenotype toward inanimate objects (34). This phenomenon can additionally be observed with aging in *Shank3b* ^+/+^ and *Shank3b^+/-^* mice. Overall, these results suggest that, at the behavioral level, *Shank3b^-/-^* mice may age faster than *Shank3b* ^+/+^ and *Shank3b^+/-^* animals.

We finally aimed at assessing whether a relationship could be found between pro-inflammatory molecules in the BM, spleen, PB, cerebellum, and motor behavior of mice (**Tables 1**-**4**). Overall, negative correlations were described between expression of pro-inflammatory molecules in peripheral organs and parameters measured in the open field and rotarod tests. In line with the discriminant analysis (**Figure 4**), the strongest and most significant correlations were present in the BM of old mice. In line with the previously described results showing a reduction of pro-inflammatory molecules in *Shank3b^-/-^* mice (**Figure 1**), positive correlations between pro-inflammatory cytokines in the cerebellum and parameters of motor test could also be documented. Our study was focused on pro-inflammatory molecules only, and the impact of anti-inflammatory responses on ASD-related behaviors was not considered. This should be assessed in future studies.

Taken together, our work suggests that a connection may exist between systemic inflammation and motor behavior in the *Shank3b* model. It is indeed interesting to note that cerebellum and spleen are the two organs that show the highest expression level of *Shank3* (https://genome.ucsc.edu/). In agreement with these findings, we showed that inflammation was associated with motor dysfunction in *Shank3b^-/-^* mice. Thus, we postulate that inflammaging is responsible for motor impairment in *Shank3b* mutants. Strategies of intervention aiming at reducing systemic inflammation might help in counteracting motor dysfunctions in ASD patients, and eventually in other pathological conditions.

## Materials and methods

### Animals

Mice were housed in a 12 h light/dark cycle with food and water available ad libitum, and kept at 25°C room temperature in the animal facility of the Center for Mind/Brain Sciences (CIMeC), University of Trento. Tested mice were single-caged two weeks before the starting of behavioral tests. Age-matched male and female *Shank3b^+/+^*, *Shank3b^+/-^*, and *Shank3b^-/-^* adult (3-5 months old; weight = 22 - 34 g) and old (>18 months old; weight = 25-51 g) mice obtained from heterozygous matings were used. All efforts were made to minimize animals’ suffering during the experiments. Numbers of mice used for each experiment are shown in the Figure legends.

### Ethical regulations

All experimental procedures were approved by the Animal Welfare Committee of the University of Trento (Organismo Preposto al Benessere Animale – OPBA) and by the Italian Ministry of Health (protocol n.922/2018-PR and n. 547/2021-PR), in accordance with the Italian law DL 26/2014 and the European Community Directive 2010/63/EU.

### Behavioral tests

Adult and old *Shank3b^+/+^*, *Shank3b^+/-^*, and *Shank3b^-/-^* mice were tested with the open field (OF), rotarod and marble burying tests. Habituation phases and behavioral experiments were performed during the light phase of the circadian cycle. Each animal was transferred to the testing room approximately 30 minutes before the starting of the tests. For all behavioral tests, male and female mice were habituated and tested separately to avoid experimental noise. Additionally, the tests’ apparatuses were cleaned with 70% ethanol to mask olfactory cues between each behavioral session.

#### Open field test

Open Field test was performed to assess general locomotor activity (36). Animals were placed in a standardized empty open field arena (40cm X 40cm X 40cm) with grey-colored walls and allowed to freely explore it for 10 minutes. Sessions were recorded by an overhead camera placed over the arena and mice were automatically video tracked using the software EthoVisionXT (Noldus). Average speed and distance moved in the arena were analyzed.

#### Rotarod test

Rotarod test was performed to assess cerebellar-associated motor coordination (25). Mice were subjected to two consecutive days of habituation sessions followed by an experimental phase on the 3rd day. The habituation phase was conducted at constant speed of 4 rotation per minute (rpm) over a 5-minute session. The experimental phase consisted in 3 trials during which the rotation speed increased from 4 rpm to 64 rpm. Falling mice landed on a metallic platform connected to a timer reporting the time spent on the rotating rod. The average time spent on the rod across three trials (latency to fall or complete 3 spins around the rod) was calculated and used as quantitative indicators of the motor ability of the mice to stay balanced on the rotating accelerating rod.

#### Marble burying test

Marble Burying test was performed to investigate engagement in repetitive behaviors, as well as object-oriented perseveration (26, 27). Mice were single placed in a standard cage (40cm × 38cm) containing bedding at a depth of ~3cm with 12 identical black marbles (15mm in diameter) arranged in a shape of a grid (3×4) on top of the bedding. After 30 minutes from the starting of the experiment, the marbles burying index was assessed by scoring 1 point for fully and partially covered marbles and 0 point for no-covered marbles.

### Tissue harvesting

Animals were sacrificed by beheading and cerebella, PBMCs, BM, and spleen cells were collected for quantitative RT-PCR (qRT-PCR) and flow cytometry assays. Cerebellar tissues used for qRT-PCR were dissected and frozen in dry ice. Samples used for flow cytometry were homogenized with pestles immediately after dissections and single-cell suspension were generated using 70 μm cell strainer (Corning). Three hundred μl of PB was harvested from each mouse and collected in a heparinized tube. PBMCs were isolated using a Ficoll-Hypaque density gradient (Sigma-Aldrich). BM cells were isolated by flushing femurs and tibias with PBS. Spleen samples were smashed through 70 µm cell strainers. After the isolation, PBMCs, BM, and spleen cells were washed once with RPMI 1640 (Sigma-Aldrich) and resuspended in complete medium (RPMI 1640 supplemented with 10% fetal calf serum, FCS, 100 U/mL penicillin and 100 μg/mL streptomycin; Sigma-Aldrich and Invitrogen respectively). In parallel, approx. 5-10x10^^6^ BM and spleen cells were lysed and stored at -80°for qRT-PCR studies.

### RNA isolation and qRT-PCR

Total RNAs were extracted from cerebella, BM and spleen cells using RNeasy Plus Mini Kit (Qiagen), quantified using NanoDrop™ Spectrophotometer (Thermo Fisher Scientific). In all samples, absorbance (A) 260/280 nm and A260/230 nm >2.0 were detected. RNA samples were retro-transcribed to cDNA using SuperScript™ VILO™ cDNA Synthesis Kit (Invitrogen™ - Thermo Fisher Scientific). qRT-PCR was performed in a CFX384™ Real-Time System (Bio-Rad), using the PowerUp™ SYBR™ Green Master Mix (Thermo Fisher Scientific). Primers (Eurofins Genomics) were designed on different exons to avoid amplification of genomic DNA. Primer sequences used for the study are shown in **Supplementary Table S1**. CFX3 Manager 3.0 (Bio-Rad) software was used to perform expression analyses. Mean cycle threshold (Ct) values from triplicate were calculated for each gene of interest and the housekeeping gene β actin, then corrected for PCR efficiency and inter-run calibration. The expression level of each mRNA of interest (normalized against β actin) was compared from triplicate experiments performed on RNA pools from 8 samples per group.

### Flow cytometry

Flow cytometry was used to quantify cytokine levels in the cerebellar, PB, BM, and spleen samples. Immunofluorescence surface staining was performed by adding a panel of directly conjugated antibody to freshly prepared cells. To assess the expression of cytokines, cells were incubated with 30 ng/mL phorbol 12-myristate 13-acetate (PMA) and 500 ng/mL ionomycin in the presence of 10 mg/mL brefeldin A (BFA) (all molecules from Sigma-Aldrich) for 4 h at 37°C. After surface staining, cells were permeabilized using the Cytofix/Cytoperm kit (BD Biosciences), and afterwards intracellular staining was performed. Labeled cells were measured using a LSR Fortessa (BD Biosciences) flow cytometer available at the Institute for Biomedical Aging Research, University of Innsbruck. Data were analyzed using Flowjo software. The antibodies used in the experiments are shown in **Supplementary Table S2**.

### Discriminant analysis

For dimensionality reduction of the FACS data we performed a discriminant analysis using the commercially available software JMPpro17. This allowed us to identify cytokines expression pattern for each of the six experimental group, using the experimental conditions as a predictor. We leveraged the average distance of each subject from the center of mass (mahalanobis distance) of each cluster to run a two-way ANOVA aimed to determine whether the cluster belonging to each of the experimental group could be considered statistically dissimilar to the other groups. The average distance of each cluster to its own center of mass was used as a reference point for comparison.

### Statistics

Statistical analyses were performed with GraphPad Prism 9.5.0 software using two-way ANOVA followed by Tukey *post-hoc* test and Spearman correlation analyses. The level of significance was set at p ≤ 0.05.

## Data Availability

All relevant data is contained within the article. The original contributions presented in the study are included in the article/supplementary material, further inquiries can be directed to the corresponding author/s.

## Supplementary material

Supplementary Figure 1 Gating strategy used for the flow cytometry experiments. Gating strategy used to define **(A)** T cells and CD14^+^ cells within cerebellar cells, and **(B)** T cells (CD8^+^ and CD4^+^), and monocytes (CD14^+^ cells) within PB lymphocytes.

Supplementary Figure 2 The expression pattern of pro-inflammatory cytokines varies with age, according to a genotype- and tissue- dependent logic. Heatmaps reporting the results of multiple comparisons on the mahalanobis distances of each group to the center of mass of each predicted cluster (**A**, bone marrow; **B**, spleen; **C**, peripheral blood). For each row, the average distance of the subjects from the center of mass is compared against the distances of all other groups from that given cluster. See also **Figure 4** for cluster analysis.

Supplementary Table 1 Sequences of the primers used for this study.

Supplementary Table 2 Antibodies used for the flow cytometry experiments.
