# Supplementary figures and images for: Immune system dysfunction and inflammation in aging Shank3b mutant mice, a model of autism spectrum disorder
Source: Front Immunol. 2024 Sep 6;15:1447385. doi: 10.3389/fimmu.2024.1447385 (PMC11412883; doi:10.3389/fimmu.2024.1447385)

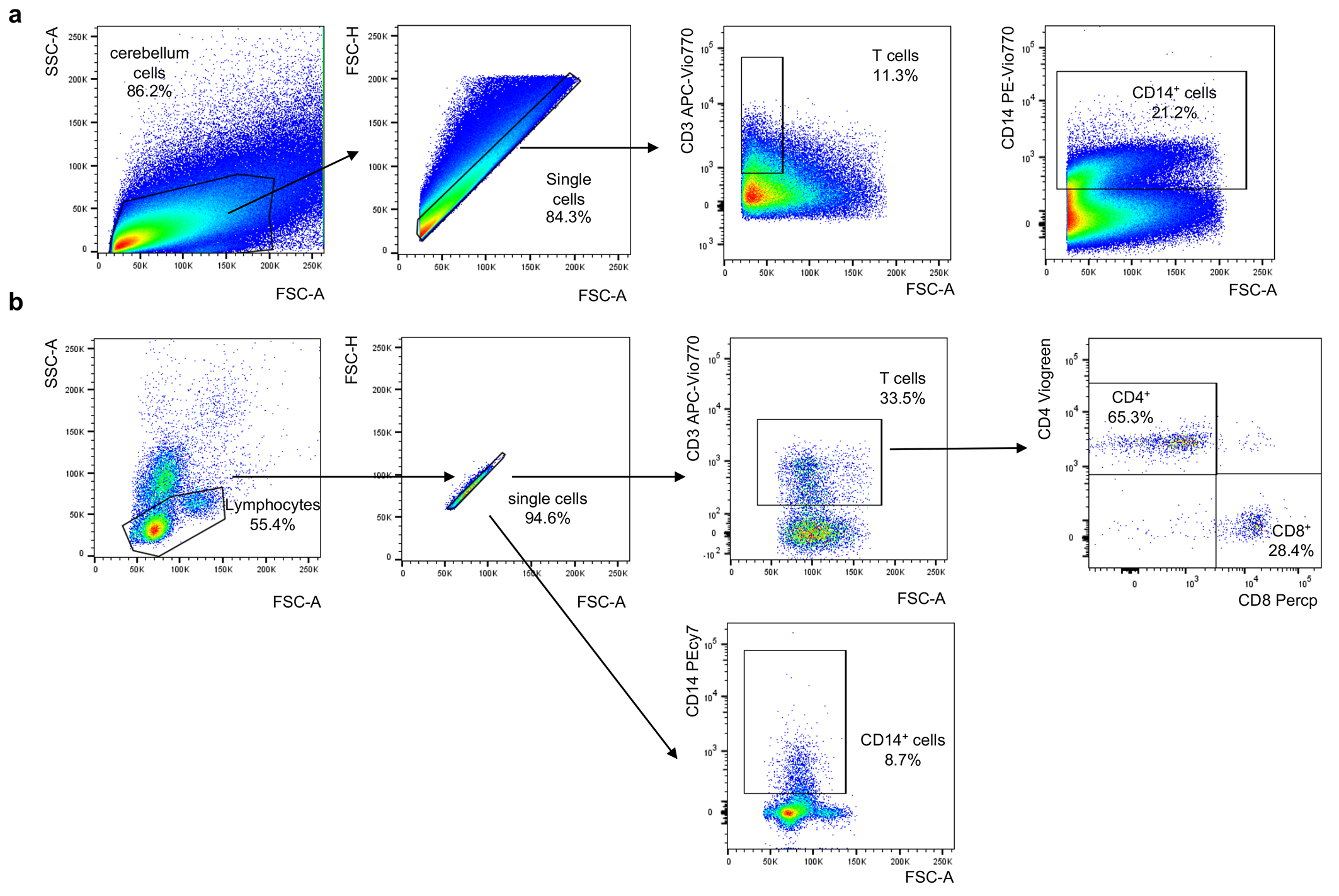

Supplement: Supplementary Figure 1 — Gating strategy used for the flow cytometry experiments. Gating strategy used to define (A) T cells and CD14+ cells within cerebellar cells, and (B) T cells (CD8+ and CD4+), and monocytes (CD14+ cells) within PB lymphocytes. [file Image1.tif]

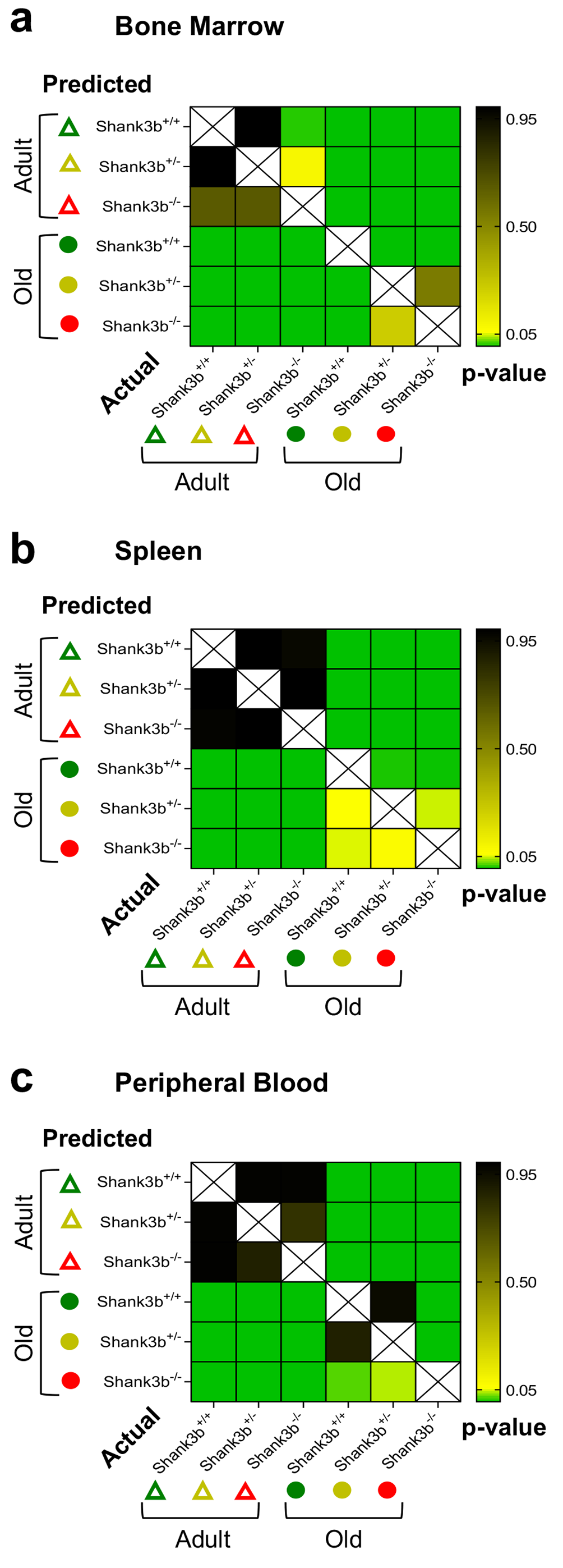

Supplement: Supplementary Figure 2 — The expression pattern of pro-inflammatory cytokines varies with age, according to a genotype- and tissue- dependent logic. Heatmaps reporting the results of multiple comparisons on the mahalanobis distances of each group to the center of mass of each predicted cluster (A, bone marrow; B, spleen; C, peripheral blood). For each row, the average distance of the subjects from the center of mass is compared against the distances of all other groups from that given cluster. See also Figure 4 for cluster analysis. [file Image2.tif]
