# Supplementary material for: Immune system dysfunction and inflammation in aging Shank3b mutant mice, a model of autism spectrum disorder
Source: Front Immunol. 2024 Sep 6;15:1447385. doi: 10.3389/fimmu.2024.1447385 (PMC11412883; doi:10.3389/fimmu.2024.1447385)
Supplement: Supplementary Table 1 — Sequences of the primers used for this study. [file Table1.docx]

**Table S1. Sequences of the primers used for this study**

| Target gene | Forward primer (5’-3’) | Reverse primer (5’-3’) |
| --- | --- | --- |
| IFNγ | CCCTATGGAGATGACGGAGA | CTGTCTGCTGGTGGAGTTCA |
| IL-6 | GCCTTCTTGGGACTGATGCT | GACAGGTCTGTTGGGAGTGG |
| MMP3 | CAGACTTGTCCCGTTTCCAT | GGTGCTGACTGCATCAAAGA |
| p21 | GACAAGAGGCCCAGTACTTC | GCTTGGAGTGATAGAAATCTGTC |
| IL-1β | ACGGACCCCAAAAGATGAAG | TTCTCCACAGCCACAATGAG |
| CCL2 | GAGTAGGCTGGAGAGCTACAAGAG | AGGTAGTGGATGCATTAGCTTCAG |
| CCL3 | TGAAACCAGCAGCCTTTGCT | AGGCATTCAGTTCCAGGTCAGTG |
| NRF2 | CTGAACTCCTGGACGGGACTA | GACAACTTGCAGCCCTTCTC |
| β actin | GGCTGTATTCCCCTCCATCG | CCAGTTGGTAACAATGCCATGT |

**Table S2. Antibodies used for the flow cytometry experiments**

| Antigen | Fluorocrome | Company | Clone |
| --- | --- | --- | --- |
| CD3 | APC-Vio770 | Miltenyi | REA641 |
| CD8 | PerCp | Biolegend | 53–6.7 |
| CD4 | VioGreen | Miltenyi | REA1211 |
| CD14 | Pecy7 | Biolegend | Sa14–2 |
| TNF | PE | Biolegend | MP6-XT22 |
| IFNγ | FITC | Miltenyi | REA638 |
| IL-6 | APC | Biolegend | MP5-20F3 |
| IL-1β | PE | Miltenyi | REA577 |
